# The Power to Detect Recent Fragmentation Events Using Genetic Differentiation Methods

**DOI:** 10.1371/journal.pone.0063981

**Published:** 2013-05-21

**Authors:** Michael W. Lloyd, Lesley Campbell, Maile C. Neel

**Affiliations:** Department Plant Science and Landscape Architecture and Department of Entomology, University of Maryland, College Park, Maryland, United States of America; Wuhan Botanical Garden, Chinese Academy of Sciences, China

## Abstract

Habitat loss and fragmentation are imminent threats to biological diversity worldwide and thus are fundamental issues in conservation biology. Increased isolation alone has been implicated as a driver of negative impacts in populations associated with fragmented landscapes. Genetic monitoring and the use of measures of genetic divergence have been proposed as means to detect changes in landscape connectivity. Our goal was to evaluate the sensitivity of Wright’s *F*_st_, Hedrick’ *G’_st_*, Sherwin’s *MI*, and Jost’s *D* to recent fragmentation events across a range of population sizes and sampling regimes. We constructed an individual-based model, which used a factorial design to compare effects of varying population size, presence or absence of overlapping generations, and presence or absence of population sub-structuring. Increases in population size, overlapping generations, and population sub-structuring each reduced *F*_st_, *G’_st_*, *MI*, and *D*. The signal of fragmentation was detected within two generations for all metrics. However, the magnitude of the change in each was small in all cases, and when *N*_e_ was >100 individuals it was extremely small. Multi-generational sampling and population estimates are required to differentiate the signal of background divergence from changes in *F_st_*, *G’_st_*, *MI*, and *D* associated with fragmentation. Finally, the window during which rapid change in *F_st_*, *G’_st_*, *MI,* and *D* between generations occurs can be small, and if missed would lead to inconclusive results. For these reasons, use of *F*_st_, *G’_st_*, *MI,* or *D* for detecting and monitoring changes in connectivity is likely to prove difficult in real-world scenarios. We advocate use of genetic monitoring only in conjunction with estimates of actual movement among patches such that one could compare current movement with the genetic signature of past movement to determine there has been a change.

## Introduction

Habitat loss and fragmentation are considered to be among the most imminent threats to biological diversity worldwide and thus are fundamental issues in conservation biology [1]–[4]. Fragmentation is a complex phenomenon that is simultaneously a consequence of habitat loss and a process in and of itself [5]–[7]. It is a function of the extensiveness of individual patches, distances among those patches [8]–[10], the nature of the intervening landscape [11], and how individual species are affected by each of those aspects [12]. Understanding the joint and independent effects of loss and configuration of the remaining habitat has long been a major focus of landscape ecology due to conservation implications e.g., [13]–[17].

Although the two phenomena are intertwined, when they are examined separately habitat loss has repeatedly been shown to have larger detrimental effects than fragmentation alone [5], [7], [18]–[21]. Still, increased isolation has been implicated as a driver of population extinctions [22], declining population size of interior species [13], [23], altered social behavior [24], reduced population viability [25], [26], demographic change in general [11], [27], [28], and spread of invasive species [29]. Reduced migration under lower levels of connectivity will have genetic consequences of reduced effective population size (*N*_e_) and increased rates of inbreeding and genetic drift within newly isolated habitat patches that will affect short- and long-term potential for survival [30]–[33].

Changes in landscape composition and configuration associated with the fragmentation process have been quantified and monitored using an extensive array of landscape indices [34]–[40]. Assessing the consequences of these changes for populations and processes fundamentally requires linking the structural attributes of landscape pattern with potential or actual movement of individuals among patches [8], [40]–[43]. Movement is often documented using habitat suitability, mark-recapture, radio-telemetry, experimental removal-recolonization studies [19], [41] and demographic monitoring [44]–[46]. Unfortunately, such studies can be so data- and time-intensive that there may be little practical application for conservation of most species e.g., [47], [48]. Observing physical movement of cryptic or primarily sessile organisms in which mobility is limited to particular life stages is especially challenging [49], [50].

Genetic monitoring [51] has been proposed as a minimally invasive, relatively cost-effective means of quantifying genetic effects of changes in landscape structure. Population genetic parameters may be more sensitive for detecting changes in connectivity than traditional demographic estimates that have large error components [52]. Thus, although in many cases conservation biologists are concerned about genetic diversity for its own sake, here we are interested in the potential for using genetic changes that result from fragmentation to quantify changes in the ecological process of movement.

Direct genetic methods have been developed to detect actual dispersal events [53]–[57]. However, it is more common for investigators to document fragmentation using indirect methods that quantify the amount of divergence in populations in putatively fragmented habitat. Although potentially more powerful analytical methods have been developed [58]–[61] and are being tested [62], [63], most investigators use Wright’s *F*_st_ [64] and its analogues [65]–[73].

Despite its fundamental importance and strong theoretical foundations, detecting genetic effects of fragmentation in the wild has not been as straightforward as one might expect. Attempts to link indices of landscape structure to ecological and evolutionary processes have not yielded consistent relationships and many empirical investigations of fragmentation fail to detect definitive effects [74]–[76]. In particular, empirical data are often equivocal relative to predictions of the impacts of fragmentation on genetic divergence. Inconsistent relationships may result from non-monotonic relationships between many landscape metrics and landscape configuration [10] or non-linear or threshold-like population responses along the fragmentation gradient. Additionally, not all habitat that is perceived as fragmented by humans is actually fragmented from the perspective of a species of interest, thus some investigations may be trying to quantify effects of fragmentation where it actually does not exist. As mentioned above, the point at which discrete patches are functionally fragmented depends on the scale at which a species perceives and interacts with the landscape [77]–[79]. For species in patchy habitats, connectivity ultimately depends on the degree to which land cover types between discrete patches are barriers, versus filters, versus easily traversable; information that is lacking for most species. Moreover, even if movement through a landscape is impeded or precluded through anthropogenic change, long-lived individuals that pre-date the fragmentation event would provide a genetic signature of connectivity that no longer exists [74]. These issues can be addressed through careful study design in which temporal and spatial sampling scales match potential scales of fragmentation based on the biology of the focal organism.

Of greater concern is the potential that characteristics of *F*_st_-related values might make them insufficient for detecting habitat fragmentation on time scales that are relevant for conservation management. Wright’s *F*_st_ and subsequent derivations have a number of specific assumptions that are almost always violated in natural systems and complicate interpretation of genetic divergence and gene flow among populations [80]–[83]. Because *F*_st_ integrates over evolutionary time it is difficult to separate current from historical processes based on a single estimate of pattern alone and it may be slow to reflect changes in migration following a fragmentation event, especially if *N*_e_ remains large. Additionally, the alleles that are most likely to be lost through drift are at low frequencies in populations and these alleles contribute little to *F*_st_ values [84]. Slow response may also arise from the fact that when connectivity is only reduced rather than eliminated entirely, estimates of *F*_st_ may remain close to zero [83]. Finally, measures of genetic structure (e.g., *F*_st_, *G*_st_, Φ_st_) can be depressed when within-subpopulation heterozygosity or variance is high relative to among-subpopulation levels, which is common with highly diverse markers e.g., microsatellites [85]–[89]. *F*_st_–related measures calculated from such data will never approach unity regardless of the underlying patterns of allelic diversity, and they do not behave monotonically. Hedrick [86] sought to overcome the dependence of *G_st_* (a generalization of Wright's *F_st_* to include multiple alleles) on levels of heterozygosity by standardizing the measure against the maximum *G_st_* possible for the observed amount of heterozygosity. The resulting statistic, *G’_st_* varies from 0–1 in a way that better reflects the underlying patterns of genetic diversity [86], but remains fundamentally based on heterozygosity. Jost [85] proposed a measure of genetic divergence based on allelic diversity (*D*) that varies between 0 and 1 regardless of within-population heterozygosity, and it is suggested to better reflect population differentiation. Heller and Siegismund [90] found that values of Jost’s *D* calculated from data in 34 published studies were ∼60% greater than the corresponding *G*_st_ values, and that *G*’_st_ values were ∼85% greater than *G*_st_. The increased magnitude of both *G’_st_* and Jost’s *D* and potential wider range of values may provide greater ability to detect recent fragmentation events. Additionally, *D* is expected to be more sensitive because it is calculated based on allele diversity which will decline more rapidly than heterozygosity [84]. More recently Sherwin has proposed a standardized mutual information (*MI*) index [91], [92] based on Shannon’s index that also varies between 0 and 1 and is independent of heterozygosity with the added property weighing all alleles according to their frequency (i.e. neither favoring rare nor common alleles).

Because we were interested in effects of fragmentation independent of habitat loss, we evaluated the ability to detect genetic effects of fragmentation with *F*_st_, *G’_st_*, *MI*, and *D* over timeframes associated with anthropogenic habitat modification (i.e., <200 years) while controlling for population size. The number of generations necessary to make such an evaluation renders the task infeasible in a field setting. Therefore, we developed an individual-based population model to simulate genetic divergence among recently fragmented populations and measured *F*_st_, *G’_st_*, *MI*, and *D* over time. Potential for detecting change in these metrics will vary based on the amount and nature of migration among populations; therefore, we simulated two severe cases of fragmentation. In the first, migration among a set of historically panmictic populations was abruptly and completely stopped. In the second, limited gene flow among populations was allowed and subsequently ceased. The first scenario provides the most ideal situation for detecting change – going from a base condition of a Wright-Fisher population to complete isolation. The second provides a more realistic starting condition in which there is a pre-existing level of divergence among populations onto which anthropogenic fragmentation is imposed. We complement a recent investigation of the effect of dispersal distance among individuals on the time required to detect an abrupt barrier to gene flow [63] by examining multiple discrete populations and by quantifying the influence of population size, overlapping generations, and sampling effort in terms of individuals and loci on ability to detect a significant change in four measures: *F*_st_, *G’_st_*, *MI*, and Jost’s *D*.

## Methods

### Model Description

We generated six homogeneous panmictic populations of equal size at the start of each run. Panmixia among populations was created by allowing mating at random among individuals in all populations. The model allows variation in distances among individual population pairs but for the purposes of this evaluation all populations were equally isolated. Census size maxima (*N*_max_) within populations were set to 25, 75, 100, 500, 1000, and 3000 individuals (*N*_e_ was subsequently calculated) which encompasses the size ranges of populations of most plant species listed under the U.S. Endangered Species Act (Neel unpublished data), and 71% of minimum viable population estimates for plant species world wide [93]. Initial size of each population was set to 75% of the size limit for each run and the size cap was reached within one or two generations.

At initiation, individuals were assigned two alleles at each of 20 unlinked microsatellite loci. Allele size ranged between 5 and 50 repeat units. Alleles for each locus could take on any value within the given range, and were drawn from a normal distribution with parameters μ = mean of the size range of the locus and σ^2^ = 5. Drawing initial allele frequencies from a normal distribution allows for accurate simulation of the stepwise mutational model of microsatellite evolution throughout a simulation [94]. These starting conditions yielded between 7 and 42 alleles per locus at the start of each simulation depending on the population size. Mutations occurred every 0.004 gamete transfer events [94]. By using a stepwise mutational model of microsatellite evolution, small changes in allelic state were more likely than large changes and the direction of mutation tended toward the mean size range of each locus [94].

Individuals were simulated to be hermaphroditic, annual plants that were self-compatible, but that did not self-fertilize more than what would be expected at random, and therefore the amount of selfing depended upon population size. All individuals had an equal probability of mating each generation. Individuals from within a population had an equal probability of being a father for all individuals within that population. The proportion of individuals contributing seed to the next generation varied around a normal distribution with the parameters μ = 50% total population size and σ^2 = ^1. The number of seeds produced per female was drawn from a normal distribution with parameters μ = 35 and σ^2^ = 5 to provide stochastic variation around a likely number of seeds per plant. Each seed had a randomly selected father. When a seed bank was included in the model, those seeds not germinating entered the seed bank; otherwise, seeds that did not germinate immediately were removed. Germination potential of seeds in the seed bank decreased over time following a negative exponential function. As the size of each population approached the population size limit, the number of viable seeds produced was reduced to reflect density dependence [95].

Each cap size was run under four conditions that independently varied presence or absence of a seed bank (i.e., non-overlapping versus overlapping generations) and presence or absence of preexisting population structure prior to population isolation. To simulate absence of population structure, panmictic populations were immediately isolated to yield an abrupt fragmentation event with the highest likelihood of being detected. To more closely reflect realistic conditions, we simulated preexisting population structure by limited seed and pollen migration as described below for 500 generations prior to stopping all migration.

At least 85% of pollen grains remained within a population and 15% had some probability of moving. Probability of dispersal from a population followed a Laplace distribution (μ = 0.4, *b* = 0), a commonly used dispersal kernel for plants that reflects a range of common dispersal syndromes [96]–[99]. Seeds produced from matings within populations could either stay within the population in which they were generated or they could disperse. Probability of dispersal followed the same dispersal kernel described above. After the dispersal step, seeds had a 10% chance of germinating the year after they were produced and their ultimate fate depended on whether or not generations overlapped. Although the specific values for seed production, seed germination, and pollen and seed dispersal were arbitrary, they were within the range of values that have been documented for plant species [100]–[106].

Simulations with preexisting population structure ran under the above conditions for 500 generations prior to complete isolation, those that began from panmixia were immediately isolated. Following isolation in both simulation types, the model proceeded for 200 additional generations with no migration among the 6 populations. We conducted 200 independent simulations for each of the four conditions for each of the six population size caps, yielding 24 model configurations. The resulting 4,800 simulations were run on The Lattice Project, a Grid computing system [107]–[110].

During simulations, individual populations were allowed to go extinct and to be recolonized with migrants from other populations (when migration was allowed) or from the seed bank (when overlapping generations were present). At small population sizes, individual populations would frequently go extinct. When all populations went extinct, the simulation was restarted. However, extinction of all six populations occurred in only ∼1/100 cases. We determined the total number of alleles, observed (*H*_o_) and expected (*H*_e_) heterozygosity at each generation.

In simulations without overlapping generations, we calculated the inbreeding *N*_e_ at each generation as 
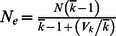
 where 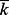 is the mean number of progeny and 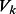 is the variance in the number of progeny at each generation [111]. In simulations with overlapping generations, *N*_e_ was calculated as *N*_e_ = *T*(*N_b_*) where *T* is generation time defined as the average age of parents including dormancy [112] calculated following Vitalis *et al*. [113] and *N*_b_ is the effective number of breeders in a given year [114]. Effective population size for each population, and for each run was calculated as the harmonic mean across all generations and then averaged across simulation runs.

At each generation we calculated Weir and Cockerham’s [115] unbiased estimate *θ*, Hedrick’s *G’*_st_ [86], Sherwin’s standardized *MI*, and Jost’s *D* [85] using the estimator *D*_est_Chao_ following Chao et al. [116]. We estimated the four measures from the total number of individuals using all 20 loci at each generation to provide the census or “true” estimate of *θ*, *G’*_st,_ *MI*, and *D*_est_Chao_ for comparison with the subsamples of individuals and loci discussed below.

We assessed the number of generations required for *θ*, *G’*_st_, *MI*, and *D*_est_Chao_ to reach equilibrium by visually assessing asymptotic behavior. We used Fisher’s exact tests to assess whether each estimated value was significantly different from 0, assuming individuals were members of a global population and then randomly reallocated to populations while maintaining sample sizes at the realized values, and recalculating each statistic [117]. The actual value for each run was compared with the distribution of 2000 such randomizations to obtain a p-value. The number of generations after population isolation at which *θ*, *G’*_st_, *MI*, and *D*_est_Chao_ became significantly different from values at the last time-step with gene flow was tested using a one-way Dunnet multiple mean comparison test in R v2.14.1 [118]. To determine the power to detect differences we calculated the proportion of runs at each generation that was significantly different from 0. The magnitude and rate of change between consecutive generations was calculated for the first 24 generations following fragmentation for all simulations.

We sampled factorial combinations of 10, 15, and 20 loci, and 20, 30, and 50 individuals (as allowed by total maximum population sizes) at every generation over the course of each simulation run. To evaluate the effect of sample size on potential to detect fragmentation, we compared estimates of *θ*, *G’*_st_, *MI*, and *D*_est_Chao_ calculated for all factorial combinations of individuals and loci to the corresponding census value using a Tukey multiple comparison test in R. In addition, we tested estimates of each measure from all factorial combinations for significant departure from 0 using the methods described above.

## Results

### All Individuals and Loci

**Figure 1:**
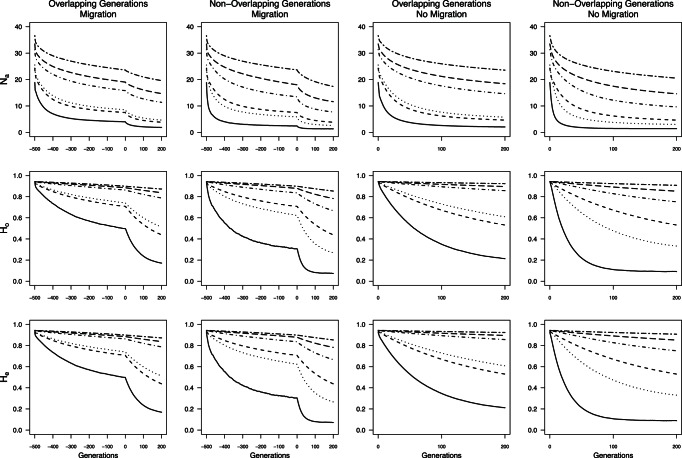
Values of N_a_, H_o_, and H_e_ for 20 loci and all individuals across all simulation conditions. Lines from top to bottom represent the *N*_max_’s of 3000, 1000, 500, 100, 75, and 25 individuals.

As expected, the number of alleles, *H*_o_ and *H_e_* tended to be higher through time in larger populations (Figure 1). Model runs with overlapping and non-overlapping generations yielded similar average allelic diversity for any given *N*_max_ (2–42 alleles per locus). However, model runs with overlapping generations tended to yield higher average *H*_o_ and *H_e_* through time than did runs with non-overlapping generations, and differences were more pronounced at smaller *N*_max_ (Figure 1).

In absence of overlapping generations, the harmonic mean values of *N*_e_ estimates for each of the six subpopulations based on all individuals averaged over all runs were 13, 40, 52, 265, 531, 1601 individuals. These *N*_e_ values represented roughly half the actual *N*_max_ values of 25, 75, 100, 500, 1000, and 3000, respectively. With overlapping generations, the harmonic mean of *N*_e_ estimates for each subpopulation averaged over all runs was roughly twice the *N*_max_: 43, 143, 193, 975, 1994, 5994 individuals, respectively.

**Figure 2:**
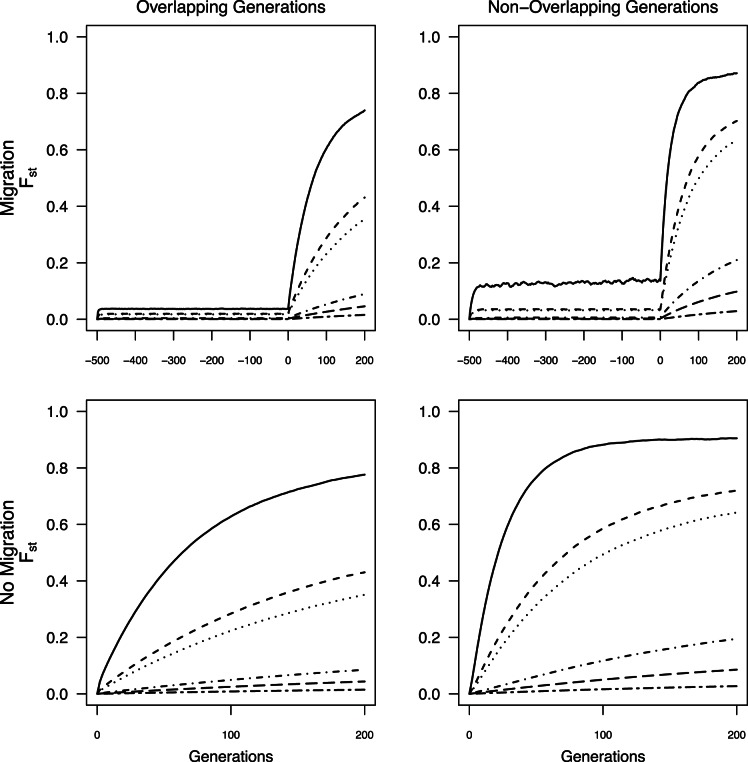
Average *θ* calculated from all individuals through time for all *N*_max_ sizes. Negative generations indicate generations with migration prior to the fragmentation event.

**Figure 3:**
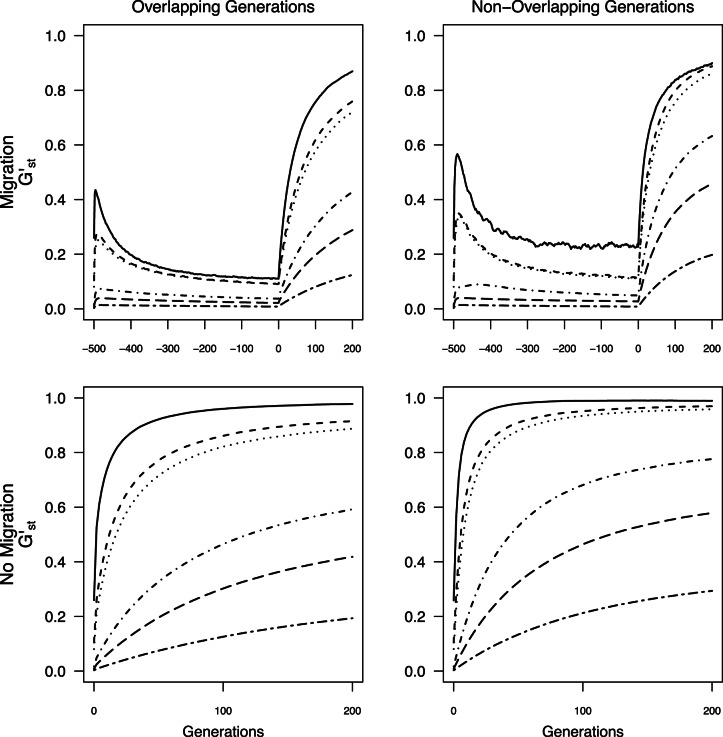
Average *G’_st_* calculated from all individuals through time for all *N*_max_ sizes. Negative generations indicate generations with migration prior to the fragmentation event.

**Figure 4:**
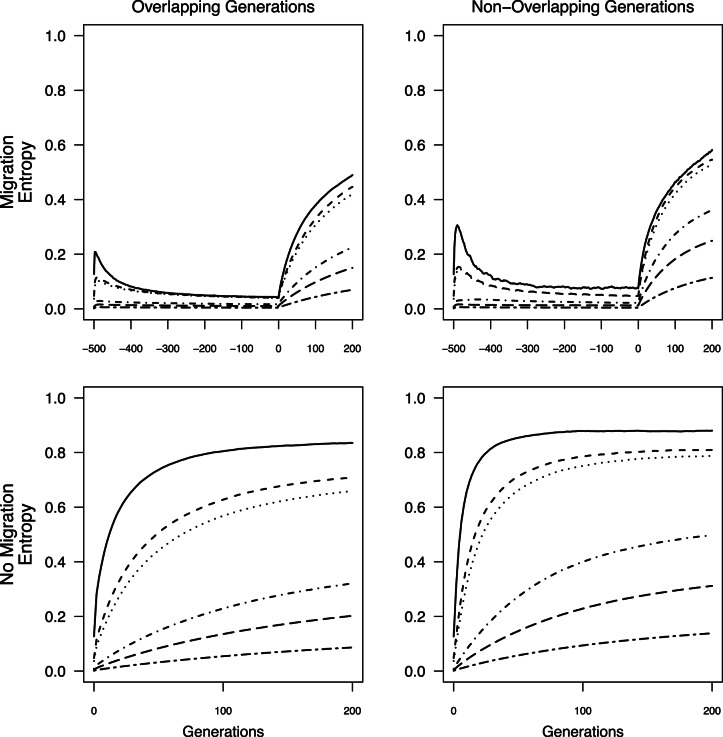
Average *MI* calculated from all individuals through time for all *N*_max_ sizes. Negative generations indicate generations with migration prior to the fragmentation event.

**Figure 5:**
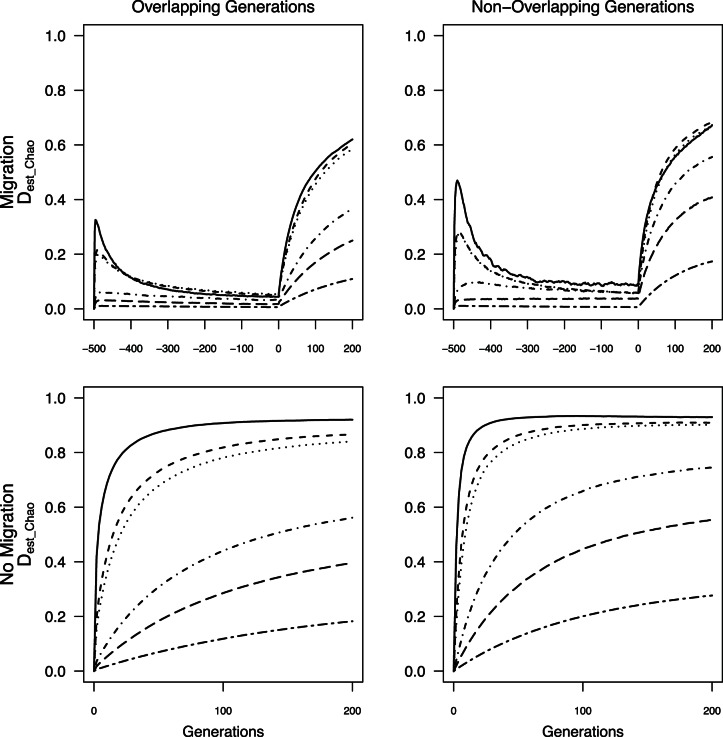
Average *D_est_Chao_* calculated from all individuals through time for all *N*_max_ sizes. Negative generations indicate generations with migration prior to the fragmentation event.

As expected from theory, behavior of *θ*, *G’*_st_, *MI*, and *D*_est_Chao_ at a given time point depended on three factors: *N*_max_, presence or absence of overlapping generations, and presence or absence of population sub-structuring prior to fragmentation. Smaller *N*_max_ predictably yielded larger values for any given time step (Figures 2, 3, 4, 5) except for *D*_est_Chao_ when *N*_max_ = 25 and generations did not overlap. For a given *N*_max_, measures were most often lower in simulations with overlapping generations than those without (Figure 2, 3, 4, 5). In simulations with population sub-structuring prior to fragmentation, *θ* and *G’_st_* values followed similar trajectories to those in which isolation occurred immediately after a period of panmixia (Figures 2 & 3). *D*_est_Chao_ and *MI* values after isolation were lower when prior population sub-structuring was included whereas *θ* and *G’_st_* were of similar magnitude (Figures 4 & 5).

Across all simulations, values of *G’*_st_, *MI*, and *D*_est_Chao_ were generally larger than *θ* under the same conditions when there was no limited migration prior to isolation (Figures 2, 3, 4, 5). When population sub-structuring preceded fragmentation and generations did not overlap, the magnitudes of *MI* and *D_est_Chao_* were lower than *θ* for *N*_max_ = 25, across all 200 generations of isolation both with and without overlapping generations (Figures 4 & 5). When *G’_st_, MI,* and *D_est_Chao_* were calculated for *N*_max = _25 with non-overlapping generations and population sub-structuring, it had a similar rate of increase to *N*_max_ = 75 and *N*_max_ = 100 under the same conditions (Figure 3, 4, 5). We found two additional anomalies: a small peak in *G’*_st_, *MI*, and *D*_est_Chao_ existed at the start of simulations that included migration when *N*_max_ ≤100 individuals (Figure 3, 4, 5).

An asymptote in values of all four measures is expected as mutation-drift equilibrium is reached [85], [86]. For *θ*, this asymptote was not reached during the 200 generation model runs when generations overlapped (i.e., with or without prior migration; Figure 2). For simulations without overlapping generations (with or without prior migration, *θ* values reached equilibrium after 60 generations when *N*_max_ = 25 individuals, and approached equilibrium by the 200^th^ generation, when *N*_max_ = 75 or 100 individuals (Figure 2). *G’*_st,_ *MI*_,_ and *D*_est_Chao_ failed to reach equilibrium with either overlapping or non-overlapping generations when there was prior migration, but rapidly did so for *N*_max_ ≤100 when isolation occurred from panmixia (Figures 3 & 5). Thus, when *N*_max_ ≥500 individuals, there was no asymptote in *θ*, *G’*_st_, *MI,* or *D*_est_Chao_ values within time scales that would affect monitoring of anthropogenic landscape change, under any of the simulation conditions.

When calculated using all loci and individuals, it took two generations after cessation of gene flow for all measures to become significantly different from zero in runs starting from panmixia and from the magnitude at the final time step with migration in the runs with pre-existing structure (Table 1). For the four combinations of pre-existing structure versus panmixia and overlapping versus non-overlapping generations, the magnitude of *θ*, when it became significant following the fragmentation event, was between 3.4×10^−4^ and 0.059. The magnitude of change in *G’_st_* at the point of significance was between 0.003 and 0.30 depending on the case. At the same time point, the magnitude of change in *MI* was between 0.003 and 0.19 and in *D*_est_Chao_ was between 0.003 and 0.47. Regardless of the simulated conditions, when *N*_max_>500 the absolute magnitude of change between generations was exceedingly small (*θ*<0.003^3^, *G’_st_* ∼0.03, *MI,* ∼0.001, *D*_est_Chao_<0.03).

**Table 1:** Difference in mean *θ*, *G’_st_*, *MI* and *D* values between the final migration step and 2 generations following cessation of migration for 200 runs under each set of simulation conditions based on full census of individuals and loci.

Overlapping Generations With Migration					Non-Overlapping Generations With Migration				
*N*_max_	Magnitude of Difference in *F*_st_	Magnitude of Difference in *G’_st_*	Magnitude of Difference in *MI*	Magnitude of Difference in *D*	*N*_max_	Magnitude of Difference in *F*_st_	Magnitude of Difference in *G’_st_*	Magnitude of Difference in *MI*	Magnitude of Difference in *D*
25	0.02684	0.04738	0.01904	0.03736	25	0.05911	0.1125	0.02398	0.09798
75	0.01032	0.03271	0.01494	0.02575	75	0.02019	0.04684	0.0212	0.02956
100	0.00821	0.02	0.01376	0.02344	100	0.01801	0.03811	0.02079	0.0296
500	0.002	0.01413	0.00645	0.01417	500	0.00372	0.02276	0.01062	0.01858
1000	0.00102	0.00493	0.00399	0.00866	1000	0.00157	0.01199	0.00607	0.01078
3000	0.00034	0.00309	0.00168	0.00344	3000	0.00038	0.00377	0.00203	0.00339
**From Panmixia**					**From Panmixia**				
***N*** **max**	**Magnitude of Difference in ** ***F*** **_st_**	**Magnitude of Difference in ** ***G’_st_***	**Magnitude of Difference in ** ***MI***	**Magnitude of Difference in ** ***D***	***N*** **_max_**	**Magnitude of Difference in ** ***F*** **_st_**	**Magnitude of Difference in ** ***G’_st_***	**Magnitude of Difference in ** ***MI***	**Magnitude of Difference in ** ***D***
25	0.04283	0.26758	0.15631	0.44143	25	0.04941	0.30642	0.18879	0.47872
75	0.01416	0.15476	0.06768	0.20209	75	0.01615	0.18334	0.08282	0.22671
100	0.01057	0.12654	0.05034	0.15958	100	0.01188	0.14867	0.06441	0.1763
500	0.00209	0.03125	0.0103	0.03541	500	0.00235	0.03751	0.01567	0.04035
1000	0.00104	0.01598	0.00695	0.01763	1000	0.00117	0.01927	0.00836	0.02004
3000	0.00035	0.00545	0.00256	0.00595	3000	0.00039	0.00661	0.00308	0.00674

We provide results for two generations because this was the point at which there was a significant difference from the last time step with migration. All differences were significant at *P<*0.05.

**Figure 6:**
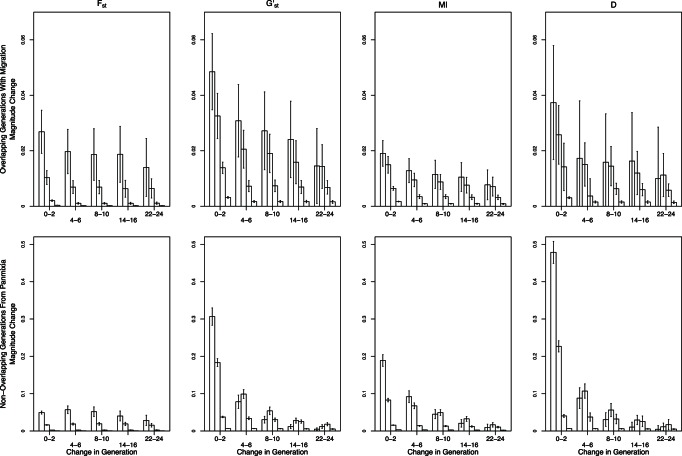
Magnitude of change between consecutive sets of two-generations over the first 24 generations following termination of migration. Bars from left to right are *N*_max_ = 25, 75, 500 and 3000 with standard error. Note the scales in the upper panel differ from those in the lower panels.

Beyond the second generation post-isolation, the magnitude of change in *G’*_st_, *MI,* and *D*_est_Chao_ between generations in the scenario with highest likelihood of detection (i.e., no overlap in generations and isolation occurred from panmixia) decreased sharply for a given *N*_max_ (Figure 6). The decline in changes in *θ* between consecutive generations was more subtle, especially through generation 10 in smaller populations, and the overall magnitude of values was much lower until generation 14–16. The effect of *N*_max_ was complicated in that changes between generations were reduced in both small populations and large populations, but for different reasons. The magnitude of change between generations when *N*_max_ = 25 was relatively constant over time and was smaller than for *N*_max_ = 75 because most populations of size 25 have already gone to fixation by the second generation, thus leaving no possibility for further divergence except through mutation. Populations with *N*_max_>75 had sufficient *N_e_* sizes to prevent substantial divergence between subsequent generations. Populations with *N*_max_ = 75 thus have the largest magnitude of change between generations until later generations when these populations also became fixed (Figure 6). Once fixation occurred within populations, the magnitude of change between generations decreased to ∼0.0005 for all measures. In the worst-case scenario for detecting change (overlapping generations and isolation from prior population sub-structure), the decline in magnitude across generations was pronounced for all four estimators and the effect of *N*_max_ was more straightforward in that they declined with increasing population size. However, the average magnitude of those changes never exceeded 0.04 and most often was <0.02 (Figure 6) and thus would be unlikely to be detected in field situations. Results for the remaining two cases, 1) generations overlapped and isolation occurred from panmixia and 2) generations did not overlap and prior population structure was included were intermediate to the presented cases (data not shown).

### Estimates from Samples

Values of *θ, MI,* and *D*_est_Chao_ calculated from samples were statistically indistinguishable from the census estimate at all time points sampled, across all simulation conditions (Tukey multiple comparison tests not shown). Thus, for *θ, MI,* and *D*_est_Chao_ the samples are unbiased and accurate estimates of the census values. In contrast, *G’_st_* for *N*_max_ = 25 was significantly larger than the census for the first 2 generations following isolation when 20 individuals were sampled, regardless of the number of loci sampled. When *N*_max_ = 75, *G’_st_* sample values were significantly larger than the census value for all generations when 20 individuals were sampled. Finally, when *N*_max_>75 values of *G’_st_* calculated from samples were significantly larger than the census value for all generations and for all sample sizes.

**Figure 7:**
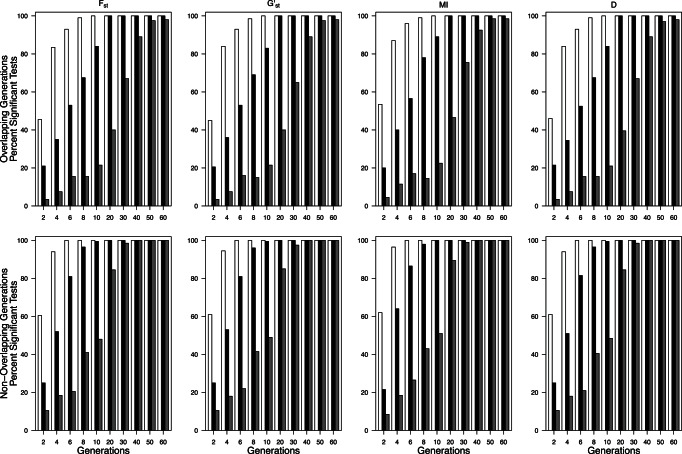
Percentage of 200 replicate runs that yielded significant *θ*, *G’_st_*, *MI,* and *D_est_Chao_* values beginning at two generations after the cessation of migration from panmixia for 20 sampled individuals and 10 sampled loci in populations overlapping generations and non-overlapping generations. Open bars *N*_max_ = 500, closed bars *N*_max_ = 1000, gray bars *N*_max_ = 3000.

**Figure 8:**
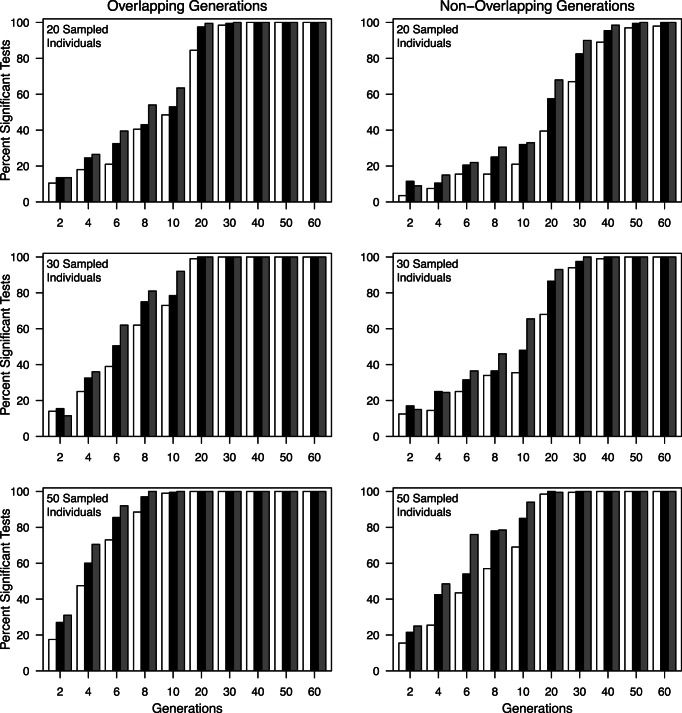
Effect of number of individuals (20, 30 and 50), number of loci (10, 15, 20), and overlapping versus non-overlapping generations on the percentage of the 200 replicate runs that yielded significant *D_est_Chao_* values 2 to 50 generations after cessation of migration for *N*_max_ = 3000. Closed bars 10 loci, open bars 15 loci, grey bars 20 loci. Data for *θ, MI,* and *G’_st_* are nearly identical and are not shown.

All sample size combinations were sufficient for detecting significant differences in *θ*, *G’*_st_, *MI*, and *D*_est_Chao_ values from 0 (when starting from panmixia), or the value prior to isolation (when prior migration was allowed) in 100% of replicates at generation 2 when *N_max_*<500 (as opposed to census values, which yielded significant differences by generation 2 at all *N*_max_ values). When *N_max_*≥500, number of individuals and loci had a large effect on power to detect differences and greatly increased the time needed to reliably detect differentiation. For example, when samples of 20 individuals and 10 loci from populations with *N*_max_ = 3000 with overlapping generations and isolation occurring from panmixia required 60 generations for 100% of samples to be significantly different from 0. For the same sample sizes, 18 generations were required when *N*_max_ = 1000, and 12 generations were required when *N*_max_ = 500. When generations did not overlap the time required for 100% of replicates to be significantly different from 0 was reduced by 50–66% (Figures 7 and 8). It took slightly longer for all samples to be significantly different from pre-isolation values when prior population structure was included (data not shown). The time required to detect a value greater than zero decreased with either larger numbers of individuals or numbers of loci (Figure 8). The addition of 10 sampled loci provided an equivalent gain to that provided by addition of 10–20 sampled individuals (Table 2).

**Table 2:** Percentage of 200 replicate runs that yielded significant *θ* values 2 generations after the cessation of migration for all factorial combinations of sampled individuals and loci.

	Overlapping Generations				Non-Overlapping Generations			
*N*_max_	Number Sampled Loci	20 Sampled Individuals	30 Sampled Individuals	50 Sampled Individuals	Number Sampled Loci	20 Sampled Individuals	30 Sampled Individuals	50 Sampled Individuals
500	10	45	80.5	98.5	10	60.5	85	99.5
	15	57.5	85.5	100	15	68	93	100
	20	73	96	100	20	85	98.5	100
1000	10	22	28	63.5	10	25	37	73
	15	26	41.5	81.5	15	31.5	49	87
	20	35.5	56.5	82.5	20	38	58	94
3000	10	3.5	12.5	15.5	10	10.5	14.5	17.5
	15	11.5	16.5	21	15	13.5	15.5	27
	20	9	15	25	20	13.5	11.5	31

## Discussion

Ideally, detecting changes in connectivity will provide early warning that biologically relevant habitat fragmentation has occurred so management action can be taken before the consequences become irreversible [51], [119]. The potential utility of indirect genetic methods for this purpose relies on a substantial and significant increase in genetic divergence following the end of migration relative to preexisting structure, as well the ability to detect that change under realistic field sampling conditions. We documented changes in *θ*, *G’*_st_, *MI*, and *D*_est_Chao_ of sufficient magnitude for detection (e.g., >0.05) under several combinations of population size and life history in our models. However, because the conditions under which changes in gene flow are likely to be detected by any of the measures were fairly restricted and because the values that could indicate fragmentation had occurred can also be obtained with natural subdivision. As such we suggest that these measures alone are likely to be problematic for confirming changes in landscape connectivity in time frames that will inform management.

On the positive side, all census estimates of *θ*, *G’*_st_, *MI*, and *D*_est_Chao_ were significantly different from 0 and from pre-fragmentation values within 2 generations of isolation when populations supported <500 individuals. This result is substantially more optimistic than that of Landguth et al. [63], which suggested that >100 generations were required for *F*_st_ to indicate fragmentation of a continuous population of 1000 individuals divided in half by a barrier to gene flow. Because of the lag time in response of *F*_st_ they recommend using Mantel’s r, which required only 1–15 generations for detection based on approaching equilibrium [63], and mention that G’_st_ responds more similarly to Mantel’s *r*, but provided no corroborating data. Because they did not report the magnitude of change in the metrics or effective population sizes, further direct or detailed comparisons with our results are not possible. Our timeframe for potential detection of significant differences was also similar to that found by England et al. [62] based on changes in *N*_e_ from a single population of 1000 that was abruptly isolated into 10 demes of 100. Our model differs from both Landguth et al. [63] and England et al. [62] in that we altered connectivity alone while maintaining population size whereas they simultaneously changed connectivity and population size.

Although we obtained significant differences within two generations, the magnitudes of the differences were often so small that detection in the field could be difficult. Magnitudes were largely dependent on the size and demography of the populations under investigation. In the best-case scenario for detecting change (*N*_max_ = 25 with no overlap in generations and isolation occurring directly from panmixia), the magnitude of *θ* two generations after isolation compared with the last generation with migration increased by 0.049 resulting in an average *θ* value of 0.04, which would be difficult to detect as biologically significant. In the same scenario, *G’_st_* increased by an average of 0.3 (resulting in an average *G’_st_* of 0.56), *MI* increased by an average of 0.18 (giving a average *MI* of 0.31) and Jost’s *D* increased on average by 0.42 (yielding an average value of 0.45) greatly increasing the potential for detection relative to *θ* estimates. In populations with ≥500 individuals, the change in *θ* from prior to fragmentation to the second generation post-fragmentation was ≤0.002, which would be viewed as biologically insignificant. Although *G’_st_, MI*, and Jost’s *D* all had larger magnitude increases for the same scenario (Table 1), detecting the increases could still be difficult. In the most difficult circumstances for detecting change (when a seed bank was present and population sub-structuring was established prior to isolation) none of the changes in *θ*, *G’*_st_, *MI,* nor *D*_est_Chao_ exceeded 0.04 within two generations, which is well within the range of sampling error in real populations [81], [120], [121]. The lower rate of change in presence of a seedbank is likely due to the doubling of the effective population size that occurred under these conditions. In total, these results indicate that detecting change from a baseline condition in two generations will be possible only when populations are <500 individuals and only when generations do not overlap.

As a practical matter, detection of changes in genetic structure due to fragmentation presumes having samples that represent conditions prior to fragmentation for comparison. It is more likely that connectivity will be assessed only after changes in habitat amount and configuration have occurred because most often species are not studied prior to becoming of conservation concern. Despite the fact that genetic monitoring by definition requires a multi-year approach to be effective [51], few published studies of fragmentation have included such temporal sampling e.g., [122]–[127], and even these efforts have generally not extended more than a few generations. Without pre-fragmentation data, it is not possible to attribute significant values of genetic differentiation measures to anthropogenic changes because such values can result from natural subdivision of smaller populations [128]. For example, *θ* values at the second generation post-isolation when *N*_max_>500 individuals and there is no prior migration, were identical to cases with limited ongoing migration when *N*_max_≤100 individuals (Figure 2). Without having precise population size estimates, it would not be possible to determine whether a given *θ*, *G’*_st_, *MI,* or *D*_est_Chao_ value reflected small population size with a low level of migration or lack of migration among larger populations.

The lack of data collected prior to landscape change can be overcome by sampling from multiple demographic cohorts representing generations that originated before and after the putative fragmentation event by, for example, examining differences between adults and more recent recruits [129] or sampling across strata in a soil seed bank [130]. Several approaches can potentially overcome lack of pre-fragmentation data when sampling demographic cohorts is not possible. Chiucchi and Gibbs [128] have suggested comparing estimates of gene flow from multiple analytical approaches that reflect different time frames as a way to compare long-term and short-term levels of differentiation from a single sample. Another approach is to compare multiple populations from similar interpatch distances in different habitat matrix types in which there is strong contrast in gene flow, or in locations with versus without barriers to gene flow, or by sampling locations at varying distances from one another [131]–[133]. Alternatively, one can sample the same populations at multiple time points after landscape change and quantify the amount of change in divergence between generations. In absence of recent change, populations are expected to be at migration drift equilibrium, at which point changes between generations will be minimal (e.g, cases with limited migration, pre isolation in Figures 2, 3, 4, 5). After fragmentation that eliminates gene flow, rapid changes towards a new equilibrium are observed. The average magnitude of change across generations exceeded change seen in absence of fragmentation or in populations with substructuring due to limited migration prior to fragmentation (Figures 2, 3, 4, 5), indicating that samples at multiple time points after isolation could allow detection of fragmentation and thus provide a solution to the lack of pre-fragmentation data. However, this signature lasts only 8–10 generations (Figure 6) when populations are ≤100 individuals; beyond this point, post-isolation the rate of change between two consecutive generations is indistinguishable from that seen in populations prior to fragmentation even though the absolute values of *θ*, *G’*_st_, *MI,* or *D*_est_Chao_ were higher than they were pre-fragmentation. In populations with >100 individuals, divergence continued increasing for the 200 generations we modeled (Figure 6), thus providing a longer temporal window for detecting changes across generations. However, when *N*_max_≥500 the magnitude of change that we observed across generations may not be large enough for detecting signatures of fragmentation in field conditions especially when generations overlap and thus a time series would be inconclusive regarding any contemporary change in genetic connectivity (Figure 5).

Additionally, for all but annual species with no seedbank, the number of years required to sample across generations could be too large to provide reasonable recommendations in timeframes that are responsive to management concerns. If generation time is 5–10 years, the 10–20 years necessary to yield a clear signal of fragmentation relative to pre-fragmentation conditions or across generations post-fragmentation does not constitute an early warning. These timeframes are also too long to be suitable for documenting if management actions have successfully reestablished connectivity in an adaptive management framework [134], which requires regular and rapid assessment of the effects of management treatments. Although we did not simulate restoration of connectivity, others have found the signature of restricted gene flow (e.g. high *F_st_*) can persist for 15–300 generations after a barrier to gene flow is removed depending on the dispersal distances [63]. A legacy of historical isolation within currently connected populations would result in misidentifying such populations as not connected by gene flow.

Should the issues surrounding sampling within the correct time window and for a sufficient length of time be overcome, the lack of power associated with sampling subsets of individuals and loci could prevent detection of changes in genetic divergence in populations of ≥500 individuals. Below that population size, sample size had no effect on the power to detect significant genetic divergence in that 100% of runs were significantly different from pre-fragmentation values. In the extreme case (*N*_max_ = 3000), when generations were overlapping it took 8 generations for at least 50% of model runs to be significantly different from zero when 50 individuals and 10 loci were sampled; this time could be reduced to 6 generations if 20 loci were sampled. In contrast, when 20 individuals and 20 loci were sampled from each population, it took more than 20 generations for 50% of runs to become significantly different from zero (Figure 8). When generations were not overlapping, these times decreased to ∼4 generations for 50 individuals and ∼8 generations for 20 sampled individuals and 20 loci (Figure 8). The tradeoffs between loci and individuals were similar to those found by England et al. [62]. Given that it is often not cost effective or feasible to obtain both additional individuals and loci, it is encouraging that both options can improve estimates. It is important to note that our recommendations apply only to use of genetic data to detect a shift in genetic connectivity and are not generalizable to all types of genetic estimates. However, our results indicate the need for sample sizes for large populations that are similar to those recommended for reliable and unbiased estimates of trends in effective population sizes (a minimum of 60 individuals, sampled at least 5 years apart, and genotyped at 15 loci [135]).

In general, over the first few generations after isolation we found that *D*_est_Chao_ *MI,* and *G’_st_* represented genetic divergence more rapidly than did Wright’s *F*_st_ across all simulation conditions. This is not that surprising given that these three measures avoid biases related to high sample heterozygosity [85]–[87] in that *D*_est_Chao_ and *MI* are calculated directly from allele frequencies and *G’_st_* controls for maximal observed heterozygosity. Although there has been disagreement surrounding the appropriateness of use of *D*_est_Chao_ to the exclusion of heterozygosity-based measures [136], [137], it has been shown to behave appropriately across a wide range of allele diversities, heterozygosities, and mutation rates [85], [88], [138]. *G’_st_* and *D*_est_Chao_ generally had a higher magnitude of change compared to *θ*, and higher overall values, except for *D*_est_Chao_ when there was prior population structure occurred and *N*_max_ = 25. *MI* was consistently lower than *G’_st,_* and *D*_est_Chao_, but was lower than *θ* when prior population structure was included in the model and when *N*_max_ <100. The equilibrium value of *MI* when generations did not overlap and isolation occurred from migration was also lower than the equilibrium *θ* value. During the initial 70 generations when migration was occurring and *N*_max_ ≤100, there was a peak in *G’_st_, MI,* and *D*_est_Chao_, which resulted from drift overwhelming migration, or from the initial increase in the number of individuals as the population cap size is reached.

Estimates of *θ* and *G’_st_* exceeded *D*_est_Chao_ when *N*_max_ was small (e.g., *N*_max = _25) and migration was present prior to isolation. The combination of small population size and migration lead to fixation of common alleles in several populations. The pattern of fixation is what subsequently resulted in inflation of *θ* and *G’_st_* relative to *D*_est_Chao_. Because Wright’s *F*_st_ and *G’_st_* are based on heterozygosity, which does not account for particular allelic states, identical alleles that are fixed within multiple populations do not contribute to within-population heterozygosity and thus contribute to inflation of values of *θ* and *G’_st_* are unable to account for the shared alleles and is therefore artificially high (Table 3). The magnitude of *θ* and *G’_st_* will be a function of the number of fixed alleles, but in all such cases *θ* and *G’_st_* are misrepresenting the underlying pattern of differentiation, and are consequently over estimating the degree of genetic differentiation relative to *D_est_Chao_*.

**Table 3:** Example cases of allelic composition drawn from *N*_max_ = 25, which included population sub-structuring; values calculated for *θ*, *G’_st_*, and *D_est_Chao_* from these sample data.

F_st_	0	1	1	1	1
G’_st_	0	1	1	1	1
D	0	0.5	0.6	0.8	1
Pop 1	A/A	A/A	A/A	A/A	A/A
	A/A	A/A	A/A	A/A	A/A
	A/A	A/A	A/A	A/A	A/A
Pop 2	A/A	A/A	A/A	A/A	B/B
	A/A	A/A	A/A	A/A	B/B
	A/A	A/A	A/A	A/A	B/B
Pop 3	A/A	A/A	B/B	B/B	C/C
	A/A	A/A	B/B	B/B	C/C
	A/A	A/A	B/B	B/B	C/C
Pop 4	A/A	B/B	B/B	C/C	D/D
	A/A	B/B	B/B	C/C	D/D
	A/A	B/B	B/B	C/C	D/D

The fixation of common alleles removes all heterozygosity and results in inflated estimates of genetic differentiation when using *F*_st_ and *G’_st_* as opposed to *D*.

Our results from samples show that even relatively few sampled individuals (20) or loci (10) provided unbiased estimates of *θ*, *MI*, and *D*_est_Chao_. In contrast, when population sizes were ≥100, estimates of *G’_st_* were always significantly larger than the census values regardless of the number of sampled individuals or loci. As proposed by Hedrick [86], we calculated *G’_st_* based solely on measures of heterozygosity, and did not include any adjustments for differences in population size. Without controlling for population size, it is not surprising that the values of *G’_st_* were biased relative to the census values. It is possible to calculate a corrected fixation index that accounts for bias that arises when from sampling a limited number of populations [139]. However, the method we used to calculate *G’_st_* is the formulation commonly calculated in population genetic software e.g., SMOGD [140]; GenoDive [141], is what was originally proposed by Hedrick [86], and was the formulation used in comparison of metrics conducted by Heller and Siegismund [90].

To conclude, we find that use of *F*_st_-related statistics, *G’_st_*, *MI*, or *D* for detecting and monitoring changes in connectivity among discrete populations is problematic in many real world scenarios. The conditions under which these indirect methods can best be applied include when populations support between 75 and 500 individuals, when sampling is done across multiple generations, and estimates of population size are available to allow distinguishing of the signal of background differentiation from changes associated with the loss of genetic connectivity. This multi-generation sampling must occur within the window during which rapid change in the estimators is occurring to yield conclusive results. Unfortunately, the number of years required to span a sufficient number of generations to detect a change may preclude utility. For these reasons, we caution against using indirect techniques alone for detection of fragmentation events, and advocate their use only in conjunction with direct estimates of actual movement among patches such that one could compare current movement with the genetic signature of past movement to determine that there has been a change.
